# A preoperative ultrasound-based protocol for optimisation of fluid therapy to prevent early intraoperative hypotension: a randomised controlled study

**DOI:** 10.1186/s13741-023-00320-4

**Published:** 2023-06-27

**Authors:** Marcell Szabó, András Péter Pleck, Sándor Árpád Soós, Bánk Keczer, Balázs Varga, János Széll

**Affiliations:** 1 Department of Surgery, Transplantation and Gastroenterology, Semmelweis University, Budapest, Hungary; 2 Semmelweis University, 26 Üllői Út, 1085 Budapest, Hungary

**Keywords:** Hypotension, Anaesthesia, General, Echocardiography, Ultrasound, Perioperative

## Abstract

**Background:**

Intraoperative hypotension is a risk factor for postoperative complications. Preoperative dehydration is a major contributor, although it is difficult to estimate its severity. Point-of-care ultrasound offers several potential methods, including measurements of the inferior vena cava. The addition of lung ultrasound may offer a safety limit. We aimed to evaluate whether the implication of an ultrasound-based preoperative fluid therapy protocol can decrease the incidence of early intraoperative hypotension.

**Methods:**

Randomised controlled study in a tertiary university department involves elective surgical patients of ASA 2–3 class, scheduled for elective major abdominal surgery under general anaesthesia with intubation. We randomised 40–40 patients; 38–38 were available for analysis. Conventional fluid therapy was ordered on routine preoperative visits. Ultrasound-based protocol evaluated the collapsibility index of inferior vena cava and lung ultrasound profiles. Scans were performed twice: 2 h and 30 min before surgery. A high collapsibility index (≥ 40%) indicated a standardised fluid bolus, while the anterior B-profile of the lung ultrasound contraindicated further fluid. The primary outcome was the incidence of postinduction and early intraoperative (0–10 min) hypotension (*MAP* < 65 mmHg and/or ≥ 30% of decrease from baseline). Secondary endpoints were postoperative lactate level, urine output and lung ultrasound score at 24 h.

**Results:**

The absolute criterion of postinduction hypotension was fulfilled in 12 patients in the conventional group (31.6%) and 3 in the ultrasound-based group (7.9%) (*p* = 0.0246). Based on composite criteria of absolute and/or relative hypotension, we observed 17 (44.7%) and 7 (18.4%) cases, respectively (*p* = 0.0136). The incidence of early intraoperative hypotension was also lower: HR for absolute hypotension was 2.10 (95% *CI* 1.00–4.42) in the conventional group (*p* = 0.0387). Secondary outcome measures were similar in the study groups.

**Conclusion:**

We implemented a safe and effective point-of-care ultrasound-based preoperative fluid replacement protocol into perioperative care.

**Trial registration:**

The study was registered to ClinicalTrials.gov on 10/12/2021, registration number: NCT05171608 (registered prospectively on 10/12/2021).

**Supplementary Information:**

The online version contains supplementary material available at 10.1186/s13741-023-00320-4.

## Introduction

Although intraoperative hypotension has no universal definition, a huge number of reports have confirmed its detrimental effect on postoperative morbidity and mortality (Lienhart et al. 2006; Sun et al. 2015; Wesselink et al. 2018). In a previous report, Südfeld et al. defined early intraoperative hypotension for events occurring between 1 and 30 min after induction of general anaesthesia (Südfeld et al. 2017). These complications are linked mostly to patient characteristics, the appropriacy of anaesthesia, and preoperative preload disturbances. Since propofol as an induction agent is extensive, and consequent vasoplegia is a major trigger event (Muzi et al. 1992), the identification of vulnerable patients makes risk stratification a major preoperative question. Available risk assessment models (e.g. the so-called HEART score) evaluate preoperative vital signs, patient history and data on preceding medication (Cheung et al. 2015). As most of these parameters are only partially or non-modifiable, the prevention of severe preoperative dehydration, evaluation of preload and replacement of the lost fluid have vital importance. Preoperative, focused cardiac ultrasound by the anaesthetist has been an evolving field for more than a decade (Bøtker et al. 2014; Cowie 2011). Most protocols estimate patients’ volume status, and many of such methods rely on the respiratory variations of the inferior vena cava for this purpose. Although the well-documented methods of preload assessment are less accurate estimates of the fluid status or fluid responsiveness when used on spontaneously breathing patients (Bortolotti et al. 2018; Preau et al. 2017) than in the case of mechanical ventilation (Barbier et al. 2004; Feissel et al. 2004), several studies including our previous experiences identified their potential role prior to general anaesthesia (Aissaoui et al. 2022; Fiza et al. 2020; Szabo et al. 2019; Zhang and Critchley 2016). Even though a recent study showed the superiority of variations of left ventricular outflow velocity–time integral (LVOT-VTI) in the prediction of intraoperative hypotension (Aissaoui et al. 2022), the feasibility of the evaluation of IVC collapsibility index (further IVCCI) still makes it an option to guide perioperative fluid therapy. Previous studies effectively introduced protocols aiming to prevent hypotension associated with spinal anaesthesia where high levels of IVC collapsibility indicated fluid therapy (Ceruti et al. 2018; Ni et al. 2022). No similar studies are available to date in the context of general anaesthesia. As fluid overload is also a major concern after surgery, early identification of hypervolemia is of vital importance as well (Oh et al. 2019; Silva et al. 2013). However, safety limits are rarely included in fluid-therapy protocols (Cecconi et al. 2015). Lung ultrasound can identify overhydrated patients (or patients who will not benefit from further fluid therapy) before the appearance of clinical signs of pulmonary congestion (a cornerstone of the so-called FALLS protocol: fluid administration limited by lung sonography) (Lichtenstein 2012). Consequently, a combination of preoperative IVC and lung ultrasound is a logical option to address this issue.

In the present study, we aim to evaluate the effectiveness and safety of an ultrasound-based preoperative fluid therapy protocol in the prevention of early intraoperative hypotension.

## Methods

### Study design

We performed a single-centre, randomised, controlled study. The study site was the Department of Surgery, Transplantation and Gastroenterology of Semmelweis University (Budapest, Hungary), a tertiary university unit. We included patients on predefined days between 15 December 2021 and 22 August 2022. Our protocol was reviewed and approved by the ethical board of the National Public Health Centre (NPHC) of Hungary on 08/12/2021 (chair: Cecília Müller, chief medical officer, number of approval 63,977–8/2021/EÜIG) and prospectively registered at ClinicalTrials.gov (NCT05171608).

### Patients

Subjects were ≥ 18 years, ASA 2 or 3 classified patients, scheduled for elective major abdominal surgery under general anaesthesia requiring endotracheal intubation. Major surgery was defined as laparotomy or laparoscopy for a surgical procedure with an expected duration longer than 60 min. Inclusion and exclusion criteria are detailed in Table 1. All patients were informed about the research purposes along with the practical aspects of the protocol, and written informed consents were obtained. Patient enrolment was done on a daily basis. An independent observer assessed the patients, and the first eligible patient was included in the order of the operating room schedule. For feasibility reasons, we limited the inclusions: one eligible subject per day was investigated. The ultrasound measurements were performed uniformly. Randomisation was subject of a visualisable IVC. The included patients were block-randomised into two groups in 1:1 ratio, according to a computerised random list. The allocation remained concealed to the investigators until this phase. Subsequently, patients were assigned to receive conventional fluid therapy (*CFT group*) or the ultrasound-based protocol (*USP group*) treatment.

**Table 1:** Eligibility criteria

Inclusion criteria	Exclusion criteria
Elective surgery	Emergency procedure Reoperation, redo procedure Incapacitated patient Uncontrolled hypotension (< 90 mmHg) Uncontrolled hypertension (> 180 mmHg) High-risk valvular disease (e.g. aortic stenosis) Endocrine hypertension (Conn’s syndrome, pheochromocytoma) Sepsis (infection and SOFA ≥ 2 pts.) Conditions blocking lung ultrasound (pneumothorax without drainage, former pulmonary resection, pleural effusion affecting more than 2 interspaces) Pregnancy
General surgical procedures	
Estimated duration of anaesthesia > 60 min ASA classes 2 or 3	

*ASA* American Society of Anesthesiology, *SOFA* Sequential Organ Failure Assessment

### Preoperative ultrasound

The ultrasounds were performed by an independent observer not involved in the individual patient’s care who had at least 3 years of previous point-of-care ultrasound experience and adequate institutional training. The subjects were scanned 3 times: 2 preoperative combined IVCCI and BLUE (bedside lung ultrasound in emergency) scans and a postoperative LUS (lung ultrasound score) exam were performed. The equipment consisted of a Philips InnoSight and a 2–6 MHz curved array probe (Koninklijke Philips NV, Eindhoven, the Netherlands) uniformly. All subjects were examined in a standard 30-degree semirecumbent position during normal spontaneous respiration. The IVC was visualised from subcostal long-axis view in 2D mode. Assessment of the diameter of the IVC (dIVC) and IVCCI M-mode measurements was done 1–3 cm from the atrium. The collapsibility index (IVCCI) was calculated using the following formula: (dIVC expiration — dIVC inspiration)/dIVC expiration × 100 = IVCCI. Transhepatic lateral approach was also allowed as a second choice (Valette et al. 2020). An *IVCCI* ≥ 40% was considered collapsible.

**Fig. 1:**
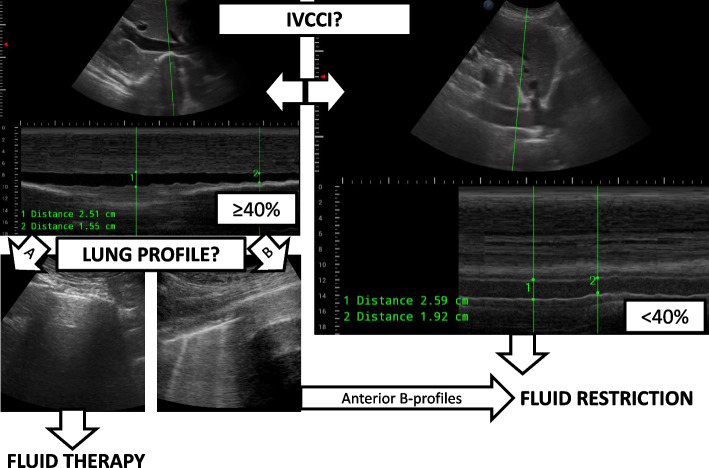
The decision tree and the typical findings of the preoperative ultrasounds

The evaluation of IVCCI was followed by a lung ultrasound exam on 3–3 BLUE points of each hemithoraces where the appropriate lung profiles (A: A-lines are predominant, B: more than 2 B-lines are detected, and C when consolidation was present or PLAPS for the presence of posterolateral pleural effusions and atelectasis) were documented as described by Lichtenstein et al. (Lichtenstein and Mezière 2008). The same probe was used, third harmonic imaging was deactivated, and care was taken to set the focus to the proximity of the pleural line. The first scan was applied 2 h (T-2 h) before the estimated start of surgery and repeated 30 min (T-30 min) before surgery. The decision tree and the typical findings of the preoperative ultrasounds are depicted in Fig. 1.

### Interventions

For the *CFT group*, the attending anaesthetist ordered preoperative fluid therapy with balanced crystalloid (Isolyte®, Fresenius Kabi) based on physical examination during the preoperative visit. The *USP group* received 8 ml/kg balanced crystalloid infusion at T-2 h when fulfilling the conditions: *IVCCI* ≥ 40% and the absence of symmetric, anterior B-profile. T-30 min scan allowed for a further 5 ml/kg fluid bolus for the same indications or for 8 ml/kg if the above-mentioned ultrasound conditions were first verified. Diluent of systemic antibiotic prophylaxis (maximum 200 ml) was used independently of the fluid therapy but was taken into account when we summarised the volume. Allocation to the groups was identified after the first ultrasound measurement: the patient’s study ID was uploaded to the digital registry, and the result of the pre-existing randomisation was verified. The patient and the anaesthesiologist both remained blinded for the treatment group. For USP patients, the dose of fluid therapy prescribed by the attending anaesthetist was modified to USP dose. The treating anaesthesiologist was not aware of the amount of the preoperative infusion, but further intraoperative therapy and the use of vasopressors (ephedrine and/or norepinephrine) were set by their own discretion when absolute hypotension criterion of *MAP* < 65 mmHg was met. We did not use pre-emptive vasopressors for study patients.

### Anaesthesia protocol

Fasting times were at least 6 h for solids and 2 h to clear fluids. Institutional ERAS (enhanced recovery after surgery) protocols of the different surgical groups were followed. Preoperative mechanical bowel preparation with osmotic laxative (MoviPrep®) was used for colorectal patients without signs of obstruction by the decision of the surgical team. These patients received carbohydrate drinks (PreOp Drink®, Nutricia™) 2 h prior to surgery. In other patient groups, no bowel preparation was applied, and oral water intake was encouraged respecting the requested fasting times. Routine premedication using alprazolam was given 1 h before surgery. Regular cardiovascular medication of the patients was maintained on their routine, except for diuretics. All patients were monitored continuously using ECG, pulse oximetry, and capnography starting at the beginning of manual ventilation. Noninvasive blood pressure monitoring by oscillometry and invasive arterial blood pressure monitoring were used according to the needs of the planned surgery and the risk level of the patient. Noninvasive measurements were obtained at 5-min intervals, and an additional measurement was obligatory 2 min after the administration of induction drugs. This step preceded the intubation of the trachea and served for the registration of postinduction vital signs. When invasive monitoring was used, an arterial cannula (20-G FlowSwitch®, Beckton Dickinson, Sàrl, Switzerland) was inserted before induction, and postinduction vital signs were registered at the same time points as above. For those cases where the arterial cannula was inserted later only after the induction, we documented the noninvasively recorded measurements for the interval of interest to maintain consistency. When epidural cannula insertion was indicated, it was inserted and tested with lidocaine while awake; 15 min later, the spreading was assessed, but top-up was not allowed until the monitoring period of primary outcome ended. For the standardised induction of general anaesthesia, we administered fentanyl (1–2 μg/kg), propofol (1.5–2 mg/kg, slow bolus over 20–30 s) and nondepolarizing muscle relaxants (rocuronium or cis-atracurium) according to age, weight and chronic organ function. Anaesthesia was maintained with sevoflurane.

### Primary endpoint and postoperative data

The primary endpoint was the incidence of postinduction and early intraoperative hypotension. Hypotension was defined as mean arterial pressure (MAP) < 65 mmHg (absolute hypotension) or as a composite of absolute and relative hypotension. Relative hypotension was diagnosed when a ≥ 30% decrease compared to baseline (immediate before induction of anaesthesia) was detected. We registered such events from the postinduction time point to 10 min after intubation. This conforms to the definition of early intraoperative hypotension (events before the 20th min) and was mostly free of the confounding effects of surgical activity (Südfeld et al. 2017), which generally starts 10 to 20 min after induction in our institution.

Collected postoperative data involved lactate (surrogate for hypoperfusion) and blood gas levels from an arterial blood sample taken in the first postoperative hour, the dose of fluid therapy, urine output of the operative day and lung ultrasound score (LUS) calculated in the postoperative 24th h. As fluid overload is a well-described risk factor of postoperative pulmonary complications (Ishikawa et al. 2022; Rock and Rich 2003), immediate postoperative pO_2_ and LUS of the 24th h after surgery were collected. Lung ultrasound is a sensitive and noninvasive way to detect any form of postoperative pulmonary complication (PPC) (Touw et al. 2019), and higher LUS levels are strongly predictive of its later developing forms (Szabo et al. 2021; Zieleskiewicz et al. 2021). The postoperative quantitative lung ultrasound score method relied on a validated protocol of assessing lung aeration optimised for perioperative purposes. Twelve lung areas (6 of both hemithoraces) were assessed, and a LUS of 0–3 points were calculated for each following the modified LUS protocol previously described by Monastesse et al. (Monastesse et al. 2017). Briefly, A-profile was scored as 0 point, and B-profile with and > 2 well-spaced lines/interspace or coalescent B-profile was registered as 1 or 2 points, respectively. For atelectasis with diameters exceeding 1 × 2 cm, 3 points were recorded. Small non-translobar subpleural consolidations with a clear pleural line were considered with 1, multiple consolidations separated by an irregular pleural line with 2 points.

Length of hospital stay was also documented for audit purposes, but the study was not powered for this variable.

### Sample size and statistics

Sample size calculations were made based on the primary outcome and the characteristics of preoperative fluid therapy. The reported incidence of early intraoperative hypotension is reported in a range of 30–51% (Aissaoui et al. 2022). We assumed that it is possible to reduce the risk to 10–25% (absolute risk). Pairs of 50 to 20 and 30 to < 10% resulted in final sample sizes of 72–78. Concerning preoperative fluid replacement, we considered a difference of 200 ml as clinically meaningful, and we estimated its standard deviation to 300 ml (based on unpublished audit data of our unit), which also made it necessary to randomise 74 patients. The planned number of subjects was selected to 80, allowing for a 5–10% dropout rate.

A per-protocol approach was planned for the statistical analysis of the outcome. Numerical data are presented as mean ± standard deviation when normally distributed (tested by Shapiro–Wilk’s *W*-test) otherwise as median and interquartile range (IQR). Hypothesis testing was performed with Student’s or Mann–Whitney *U*-test where appropriate. Categorical data are reported as number of elements and percentage (%) and tested for independence using the *χ*^2^-test or Fisher’s exact test for low expected values. For the primary outcome analysis, we performed Kaplan–Meier analysis with logrank test where time to hypotension was the variable of interest. The results of two-tailed tests are shown; the limit of statistical significance was defined as *p* < 0.05.

## Results

### Population data

**Fig. 2:**
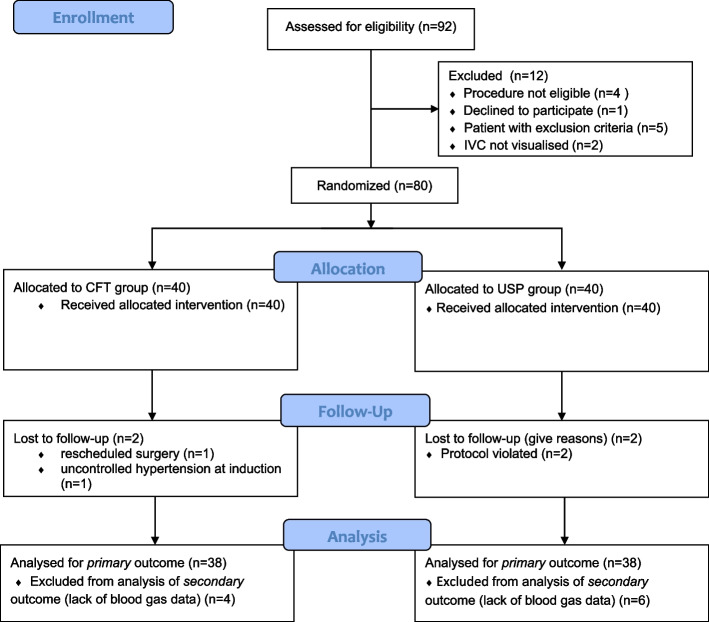
Study flowchart

Ninety-two patients were assessed for eligibility, and 80 of them were randomised. After exclusions, the data from 76 subjects were available for analysis. Reasons for exclusion at the different stages are depicted as a study flow chart in Fig. 2. Baseline characteristics of the patients and surgeries are described in Table 2. We did not find important pretreatment differences among the incidence of comorbidities or percentage of major surgical groups.

**Table 2:** Baseline characteristics

Variable	CFT group (*N* = 38)	USP group (*N* = 38)	*p*-value
Age, year, median	66 (58 to 71)	64 (52 to 73)	0.5503
Male, *N* (%)	17 (44.7%)	24 (63.2%)	0.1072
BMI, kg/m^2^	26.50 ± 5.02	27.91 ± 5.4	0.2424
ASA 3, *N* (%)	17 (44.7%)	14 (36.8%)	0.4838
Hypertension, *N* (%)	27 (71.1%)	24 (63.2%)	0.4639
Congestive heart failure, *N* (%)	7 (18.4%)	7 (18.4%)	1.0000
COPD, *N* (%)	3 (7.9%)	2 (5.3%)	1.0000
Cerebrovascular disease (past TIA/stroke), *N* (%)	2 (5.3%)	3 (7.9%)	1.0000
Creatinine, median, μmol/l	79 (66 to 101)	80 (64 to 94)	0.4619
HEART score, median (IQR)	3 (3 to 4)	3 (2 to 4)	0.0908
Preinduction systolic blood pressure, mmHg	140.9 ± 17.7	134.7 ± 23.8	0.2002
Preinduction MAP, mmHg	103.3 ± 11.6	99.9 ± 16.9	0.3119
Epidural in place, *N* (%)	13 (34.2%)	8 (21.1%)	0.1996
Propofol, mg	134 ± 26	136 ± 30	0.7466
Arterial cannula in place prior to induction, *N* (%)	8 (21.6%)	6 (15.8%)	0.5169
Surgical procedures			
*Upper GI tract, N (%)*	8 (21.1%)	10 (26.3%)	0.5895
*Hepato-pancreato-biliary, N (%)*	12 (31.7%)	6 (15.8%)	0.1055
*Colorectal, N (%)*	16 (42.1%)	21 (55.3%)	0.2512
*Other, N (%)*	2 (5.3%)	1 (2.6%)	1.0000
Laparoscopy, *N* (%)	17 (44.7%)	16 (42.1%)	0.8170
Duration of surgery, min, median	155 (118 to 240)	164.5 (120 to 220)	0.9526

*ASA* American Society of Anesthesiology, *BMI* body mass index, *CFT* conventional fluid therapy, *COPD* chronic obstructive pulmonary disease, *GI* gastrointestinal, *MAP* mean arterial pressure, *USP* ultrasound-based protocol

### Primary outcome

**Fig. 3:**
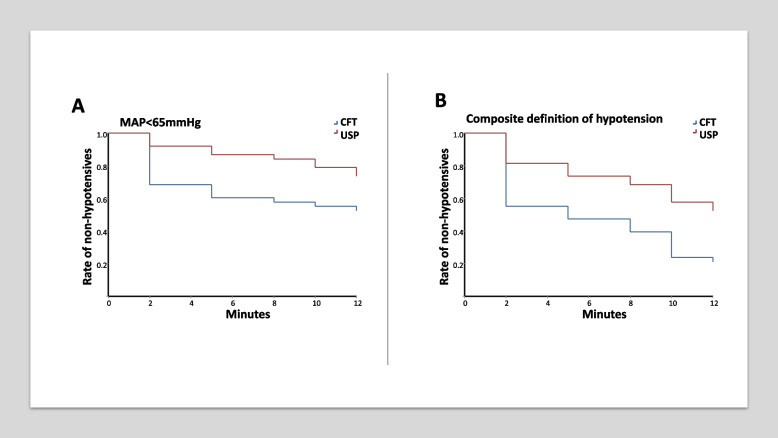
**a**–**b** The probability of maintaining normotension in the study groups based on absolute (**a**) or composite (**b**) criteria

The incidence of (immediate) postinduction hypotension is presented in Table 3. Percentage of cases when absolute or composite MAP criteria were fulfilled is shown separately. Hazard ratios of early intraoperative (including postinduction) hypotension are shown in Table 4, and the probability of maintaining normotension is depicted as Kaplan–Meier curves in Fig. 3a–b.

**Table 3:** Incidence of postinduction hypotension

Hypotensive event	CFT group (*N* = 38)	USP group (*N* = 38)	*p*-value
*MAP* < 65 mmHg	12 (31.6%)	3 (7.9%)	0.0246*
*MAP* < 65 and/or > 30% decrease	17 (44.7%)	7 (18.4%)	0.0136*

*CFT* conventional fluid therapy, *MAP* mean arterial pressure, *USP* ultrasound-based protocol

**Table 4:** Hazard ratio of early intraoperative hypotension (2–12 min), time to hypotension survival analysis

Definition	Hazard ratio	95% *CI*	*p*-value
	**CFT vs. USP as reference**		
*MAP* < 65 mmHg	2.10	1.00 to 4.42	0.0387*
*MAP* < 65 and/or > 30% decrease	2.16	1.22 to 3.82	0.0023*

*CFT* conventional fluid therapy, *MAP* mean arterial pressure, *USP* ultrasound-based protocol

Patients in the CFT group were more prone to hypotensive events than patients in the USP group. The incidence of postinduction hypotension was significantly lower when the ultrasonographic protocol was used. Regarding the cumulative frequency of early intraoperative hypertension, we detected a higher probability of maintaining normotension in the USP group using both definitions (absolute and composite) during the observational period.

### Fluid therapy

**Fig. 4:**
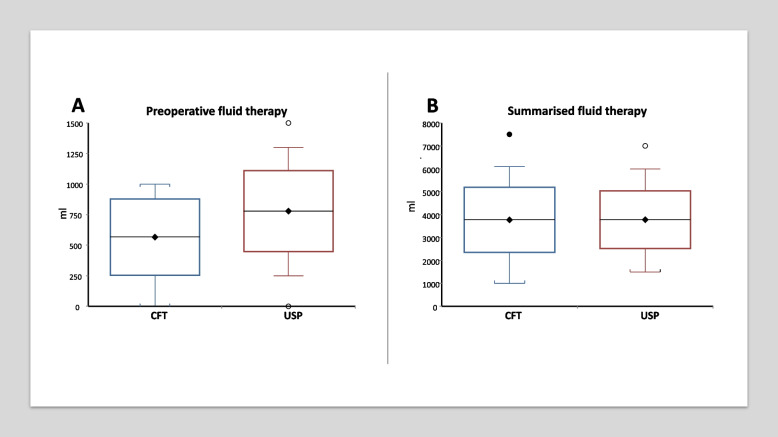
**a**–**b** Preoperative (**a**) and summarised fluid therapy of the operative day (**b**) in the study groups (mean, standard deviation and 95% CI as range)

CFT patients received significantly less fluid than USP subjects (Fig. 4a). The dose of infusion therapy was 566 ± 312 ml and 779 ± 331, respectively (*p* = 0.0051). However, the summarised fluid therapy of the operative day was not significantly different (Fig. 4b): 3773 ± 1420 ml vs. 3780 ± 1257 ml (*p* = 0.9823). Consequently, preoperative fluid represented a higher proportion of the operative day’s infusion therapy in the USP group: 14.3% (9.1 to 20.0) and 20.0% (13.3 to 18.6) (*p* = 0.0101).

### Secondary outcome: postoperative data

Immediate blood gas results were similar in the study groups. Results of arterial samplings taken in the first postoperative hour were available from 34 patients of the CFT and 32 of the USP group. Patients with lactate levels higher than 2 mmol/l were detected with a similar proportion: 6 patients (15.8%) in the CFT and 4 (10.5%) in the USP group (*p* = 0.4973).

Concerning early postoperative pulmonary complication risks, we did not experience significant differences in LUS values between the groups (Table 5).

**Table 5:** Postoperative data of the patients

Blood gas-derived variables	CFT group (*N* = 34)	USP group (*N* = 32)	*p*-value
P/F ratio, mmHg, median	392.5 (340 to 460)	393.2 (305 to 508)	0.9619
Lactate, mmol/l, median	1 (0.8 to 1.2)	0.85 (0.6 to 1.35)	0.3545
Base excess, mmol/l	− 4.75 ± 2.54	− 5.24 ± 2.0	0.3824
**Lung ultrasound on postoperative day 1**	**CFT group (*N* = 38)**	**USP group (*N* = 38)**	
LUS, median	4 (3 to 7)	4 (3 to 8)	0.8823

*CFT* conventional fluid therapy, *LUS* lung ultrasound score, P/F ratio PaO_2_/FiO_2_, *USP* ultrasound-based protocol

Operative day urine output data of 37 CFT and 36 USP patients were available for analysis corresponding to a median of 1400 ml (1100 to 1800) and 1600 ml (1200 to 1900), which did not differ significantly (*p* = 0.2256). Oliguria was encountered in 4 (10.8%) and 3 (8.3%) cases, respectively (*p* = 1.000).

Length of stay was not significantly different: 8 days (6 to 15) in the CFT and 7 days (5 to 9) in the USP group (*p* = 0.0983).

## Discussion

This study demonstrates that a combination of preoperative IVC and pulmonary ultrasound is a feasible way of guiding preprocedural fluid therapy, and it is an effective way to decrease the incidence of early intraoperative hypotension. The prerequisite of IVC visualisation made necessary only 2 exclusions, presumably not leading to considerable selection bias.

Of note, the occurrence rate of relative hypotension was relatively high, probably related to the older age of the study population and the high proportion of colorectal surgeries. Despite the encouraged oral fluid intake and carbohydrate drinks, mechanical bowel preparation was also applied frequently in this surgical group. However, the absolute MAP criterion was diagnosed in a rate which was comparable to the results of earlier studies in the CFT group, and we were able to decrease it to lower levels (Südfeld et al. 2017; Aissaoui et al. 2022; Zhang and Critchley 2016). As *MAP* < 65 mmHg is a potentially better surrogate of hypoperfusion, we assume that our protocol enabled us to promote patient safety (Wesselink et al. 2018), but a further evaluation of hard endpoints is needed.

We provided evidence that our two-step approach is safe, since it did not lead to hazardous fluid overload — as shown in the similar results in both groups’ postoperative P/F ratios and lung ultrasound scores. The observation of bilateral anterior B-profile, a safety limit in our protocol, necessitated the interruption of fluid loading only once. The beneficial effects were achieved by infusing significantly more fluid preoperatively in the treatment group, but it basically meant an earlier administration of a higher proportion of the operative day’s whole dose, which was similar in both groups.

Although all anaesthetists are aware of the well-described hazards of intraoperative hypotension, this complication is still an important issue. Despite recent advances in the implementation of enhanced recovery (ERAS) protocols (Fulop et al. 2021; Scott et al. 2015), dehydration and hypovolemia can still be common before surgery, and they are difficult to diagnose (Hahn et al. 2014; Myles et al. 2018). Albeit a large multicentric randomised controlled trial (RELIEF — Restrictive versus Liberal Fluid Therapy for Major Abdominal Surgery) queried its benefit (Myles et al. 2018), many national and ERAS guidelines promote a restrictive intra- and postoperative fluid therapy (Scott et al. 2015; Mythen et al. 2012; Pearse and Ackland 2012). Considering the potential detrimental effects of the liberal use of vasopressors, it is vital to identify dehydrated patients preoperatively. Point-of-care ultrasound was verified as a promising noninvasive method in several studies, where results offered pointing out patients with a potentially modifiable risk (Aissaoui et al. 2022; Szabo et al. 2019; Zhang and Critchley 2016; Ni et al. 2022). Despite its obvious limitations (e.g. respiratory efforts, right ventricular dysfunction, pericardial disease, higher intra-abdominal pressures) (Via et al. 2016), the measurement of IVCCI was validated as a tool to guide fluid load before subarachnoid blockade, and it effectively helped prevent hypotension (Ceruti et al. 2018; Ni et al. 2022). Similar studies for general anaesthesia have not been available yet, possibly due to issues of standardisation.

An available model relying on the characteristics of the arterial waveform (Hypotension Prediction Index algorithm on the EV1000 system) offers an excellent prediction for hypotensive events, but its use as a guide to support clinical decision-making did not reduce the risk of significant drop in blood pressure (Maheshwari et al. 2020). Clinicians had little time (4 min as average from the alarm) to respond before hypotension developed. The short timeframe can be a limiting factor especially when fluid administration is desirable. This finding underlines the importance of extending the preventive measures to the direct preoperative period when a noninvasive method like ultrasound has self-explanatory advantages.

Our secondary outcome measures were appropriate to verify the safety of our USP, but we did not detect better early postoperative organ functions. These differences are also dependent on the treatment standards of the ‘control’ group. The potentially detrimental outcomes, like postoperative oliguria or lactic acidosis, were rare in both study groups — possibly because we used fluid therapy of similar overall amounts on the operative day. A well-performed interventional study aiming to decrease the risk of renal failure by preoperative hydration used a much higher amount of infusion than we did and also failed to verify the effectiveness of their protocol (Serrano et al. 2016). The daily intravenous fluid regimens in our study can be classified ‘liberal’ as defined by a meta-analysis from Varadhan et al. (> 2.75 l per day) (Varadhan and Lobo 2010), but absolute limits of fluid therapy are less useful to distinguish restrictive and liberal protocols, especially when less attention was paid to the estimation of preoperative dehydration in some previous studies. A similar ‘liberal’ therapy in the mentioned RELIEF trial was not associated with a lower rate of disability-free survival and even decreased the risk of renal failure compared to a ‘restrictive’ protocol (Myles et al. 2018).

Our study has some obvious limitations. We performed a single-centre study limiting the generalisation of our findings. The beneficial effect of USP-guided treatment seemed time dependent, as the between-group differences were more prominent at the immediate postinduction timepoint. It could be, at least partly, explained by the lack of standardisation in the maintenance of anaesthesia and by the variability of the exact start of surgical manipulation (including patient positioning). The timeframe for registering intraoperative hypotension was 12 min at most. This allowed us not to interfere with surgical manipulations or positioning, and we did not have to postpone the start of the surgical intervention. A similar-sized study from Myrberg et al., where preoperative fluid bolus was not linked to ultrasound findings, seemed similarly effective (Myrberg et al. 2019), but the option of individualising the fluid loading in our protocol is an additive potential. A further limitation was accepted in order not to create a cumbersome protocol, and we limited the occasions of preoperative ultrasounds to two. This allowed for an easy-to-intervene evaluation, but some of the patients with subclinical hypovolemia probably remained untreated. It is also remarkable that a high proportion of our patients was noninvasively monitored. The arterial waveform monitoring could be the gold standard and allows for beat-by-beat detection and immediate treatment of hypotension (Maheshwari et al. 2018), but, in many cases, it is unnecessarily invasive. Albeit this monitoring was applied for many of our patients later during the surgical procedure, we recorded invasive measurements when they were available for the entire observation period even with at least one preinduction reading. This approach was used for a limited number of high-risk individuals with significant cardiovascular comorbidities. These patients with arterial line in situ prior to the induction were similarly represented in both study groups.

Use of epidural analgesia is another potential confounder. To minimise its effect on cardiovascular responses, we waited 15 min after placement and did not top up epidurals in the observation time of hypotension. This modality was more frequently used in the CFT group, but the difference was not significant.

These limitations can be addressed at multicentric level with more uniform monitoring method and/or involvement of stratification or minimisation factors into the randomisation process (Scott et al. 2002). Wide-scale introduction of our protocol to the daily practice is also subject of adequate training and sufficient time for preoperative assessment and optimisation. However, the learning curve of the investigated ultrasound signs is generally steep (Vignon et al. 2011; Volpicelli et al. 2012) making the area a promising field.

## Conclusion

Our results have two promising aspects: (1) the implementation of our preoperative ultrasound-based fluid replacement protocol is an effective way to help maintain hemodynamic stability after the induction of general anaesthesia, and (2) we can set the proper timing of fluid therapy.

## Supplementary Information

**Additional file 1. **Online_dataset: dataset of the study. Categorical questions were marked with 1or 0. Abbreviations used in the table headers are explained as comments.

## Data Availability

All data generated or analysed during this study are included in this published article (and its supplementary information files).

## Abbreviations

BLUE: Bedside lung ultrasound in emergency
CFT: Conventional fluid therapy
dIVC: Diameter of the inferior vena cava
ERAS: Enhanced recovery after surgery
FALLS: Fluid administration limited by lung sonography
IVC: Inferior vena cava
IVCCI: Collapsibility index of the IVC
LVOT-VTI: Velocity-time integral measured in the left ventricular outflow tract
LUS: Lung ultrasound score
PPC: Postoperative pulmonary complication
USP: Ultrasound-based protocol
